# Impaired GK-GKRP interaction rather than direct GK activation worsens lipid profiles and contributes to long-term complications: a Mendelian randomization study

**DOI:** 10.1186/s12933-024-02321-z

**Published:** 2024-06-29

**Authors:** Ke Wang, Mai Shi, Andrea O. Y. Luk, Alice P. S. Kong, Ronald C. W. Ma, Changhong Li, Li Chen, Elaine Chow, Juliana C. N. Chan

**Affiliations:** 1 Department of Medicine and Therapeutics, The Chinese University of Hong Kong, Prince of Wales Hospital, Shatin, Hong Kong Special Administrative Region China; 2 Li Ka Shing Institute of Health Sciences, The Chinese University of Hong Kong, Prince of Wales Hospital, Shatin, Hong Kong Special Administrative Region China; 3 Hong Kong Institute of Diabetes and Obesity, The Chinese University of Hong Kong, Prince of Wales Hospital, Shatin, Hong Kong Special Administrative Region China; 4 Phase 1 Clinical Trial Centre, The Chinese University of Hong Kong, Prince of Wales Hospital, Shatin, Hong Kong Special Administrative Region China; 5 Hua Medicine (Shanghai) Co., Ltd., Shanghai, China

**Keywords:** Glucokinase, Glucokinase activator, Glucokinase regulatory protein, Mendelian randomization, Triglycerides, Metabolic dysfunction-associated steatotic liver disease, Coronary artery disease

## Abstract

**Background:**

Glucokinase (GK) plays a key role in glucose metabolism. In the liver, GK is regulated by GK regulatory protein (GKRP) with nuclear sequestration at low plasma glucose level. Some GK activators (GKAs) disrupt GK-GKRP interaction which increases hepatic cytoplasmic GK level. Excess hepatic GK activity may exceed the capacity of glycogen synthesis with excess triglyceride formation. It remains uncertain whether hypertriglyceridemia associated with some GKAs in previous clinical trials was due to direct GK activation or impaired GK-GKRP interaction.

**Methods:**

Using publicly available genome-wide association study summary statistics, we selected independent genetic variants of *GCKR* and *GCK* associated with fasting plasma glucose (FPG) as instrumental variables, to mimic the effects of impaired GK-GKRP interaction and direct GK activation, respectively. We applied two-sample Mendelian Randomization (MR) framework to assess their causal associations with lipid-related traits, risks of metabolic dysfunction-associated steatotic liver disease (MASLD) and cardiovascular diseases. We verified these findings in one-sample MR analysis using individual-level statistics from the Hong Kong Diabetes Register (HKDR).

**Results:**

Genetically-proxied impaired GK-GKRP interaction increased plasma triglycerides, low-density lipoprotein cholesterol and apolipoprotein B levels with increased odds ratio (OR) of 14.6 (95% CI 4.57–46.4) per 1 mmol/L lower FPG for MASLD and OR of 2.92 (95% CI 1.78–4.81) for coronary artery disease (CAD). Genetically-proxied GK activation was associated with decreased risk of CAD (OR 0.69, 95% CI 0.54–0.88) and not with dyslipidemia. One-sample MR validation in HKDR showed consistent results.

**Conclusions:**

Impaired GK-GKRP interaction, rather than direct GK activation, may worsen lipid profiles and increase risks of MASLD and CAD. Development of future GKAs should avoid interfering with GK-GKRP interaction.

**Supplementary Information:**

The online version contains supplementary material available at 10.1186/s12933-024-02321-z.

## Background

Diabetes is a pressing global healthcare concern. Many patients have suboptimal glycemic control with high risk for multiple complications [1], calling for new treatment strategies. Glucokinase (GK) is the first and rate-limiting enzyme of glycolysis that senses glucose levels in the pancreas and liver [2]. In the pancreatic β-cells, GK phosphorylates glucose to Glucose-6-Phosphate (G-6-P), which is essential for ATP production and insulin secretion. In the liver, GK enhances hepatic glucose uptake and glycogen synthesis. Hepatic GK is modulated by glucokinase regulatory protein (GKRP), a GK regulator which is exclusively expressed in hepatocytes. GKRP binds to GK and forms a complex sequestered in the nucleus when plasma glucose (PG) and fructose levels are low to avoid excessive hepatic glucose uptake and hypoglycemia. In response to rising PG levels, GK can be rapidly released from the complex to promote glycogen synthesis [3]. Dysregulation of GK function due to genetic variants can cause abnormal glucose metabolism. For example, glucokinase-maturity-onset diabetes of the young (GK-MODY) and persistent hyperinsulinemic hypoglycemia of infancy (PHHI) are caused by inactivating and activating *GCK* mutations, respectively [4, 5]. Individuals with GK-MODY due to inactivating mutations have impaired glucose sensing with a higher setpoint which triggers increased insulin secretion which may lead to mild and asymptomatic fasting hyperglycemia (5.5–8 mmol/L) [6]. Until the recent introduction of a GK activator known to restore the glucose-sensing and insulin-secreting setpoints [7], conventional oral antidiabetic drugs (OADs) are not effective among these patients [6].

GK activators (GKAs) are a new class of OADs targeting GK since 2003 [8–12]. These compounds bind to GK, allosterically modulate enzymatic kinetics, enhance glucose sensitivity in beta-cells with improved insulin response. GKAs can be divided into liver-selective and dual-acting (liver and pancreas) activators. In addition to directly activating GK by stabilizing its active conformation, some GKAs, although not intentionally designed, can impair the protein–protein interaction of GK-GKRP complex in hepatocytes. The latter can lead to increased GK translocation from the nucleus to the cytoplasm [13, 14] with excessive hepatic glucose uptake. When excessive glucose hepatic flux exceeds the capacity of glycogen synthesis, hypertriglyceridemia may ensue due to increased glucose-fatty acid cycle [15]. This adverse effect has led to the discontinuation of development of some GKAs in the past [16]. Likewise, GK-GKRP disruptors had been developed as OADs with reduced GK nuclear sequestration and increased hepatic GK activity [17, 18]. Although GK-GKRP disruptors exhibited low hypoglycemic risk [17], potential hypertriglyceridemia due to excess liver glycogen deposition and de novo lipogenesis are significant concerns [19, 20]. Enhanced hepatic de novo lipogenesis may contribute to hepatic lipid accumulation and result in metabolic dysfunction-associated steatotic liver disease (MASLD), formerly known as non-alcoholic fatty liver disease (NAFLD), with dyslipidaemia and MASLD being both independent risk factors for cardiovascular diseases [21, 22]. With better design of the molecules, the more recently developed GKAs such as dorzagliatin (a dual-acting GKA) and TTP399 (a liver-selective GKA) [11, 23–25] did not cause hypertriglyceridemia in the clinical studies. Notably, dorzagliatin, the first-in-class GKA, has been approved in China in 2022, showing a placebo-subtracted HbA1c reduction of 0.5% in phase 3 studies in patients with type 2 diabetes (T2D) with a good safety profile [23, 24].

In our previous work, we investigated the influence of GK activation on cardiovascular disease risk using Mendelian randomization (MR) analysis and reported that target-specific glucose-lowering through GK activation might confer protection against coronary artery disease (CAD) and heart failure (HF) [26]. Importantly, the cardiovascular benefits of GK-targeted glucose-lowering exceeded that due to non-targeted glucose-lowering [26]. In the present study, we sought to discern how different GK activating mechanisms might influence long-term cardiovascular outcomes via their impacts on lipid-related traits. We hypothesized that disruption of GK-GKRP interaction, rather than GK activation per se, could worsen lipid profiles and contribute to long-term complications. We tested our hypothesis through MR analyses and corroborated our primary findings using data from a prospective cohort.

## Methods

### Study design

**Fig. 1:**
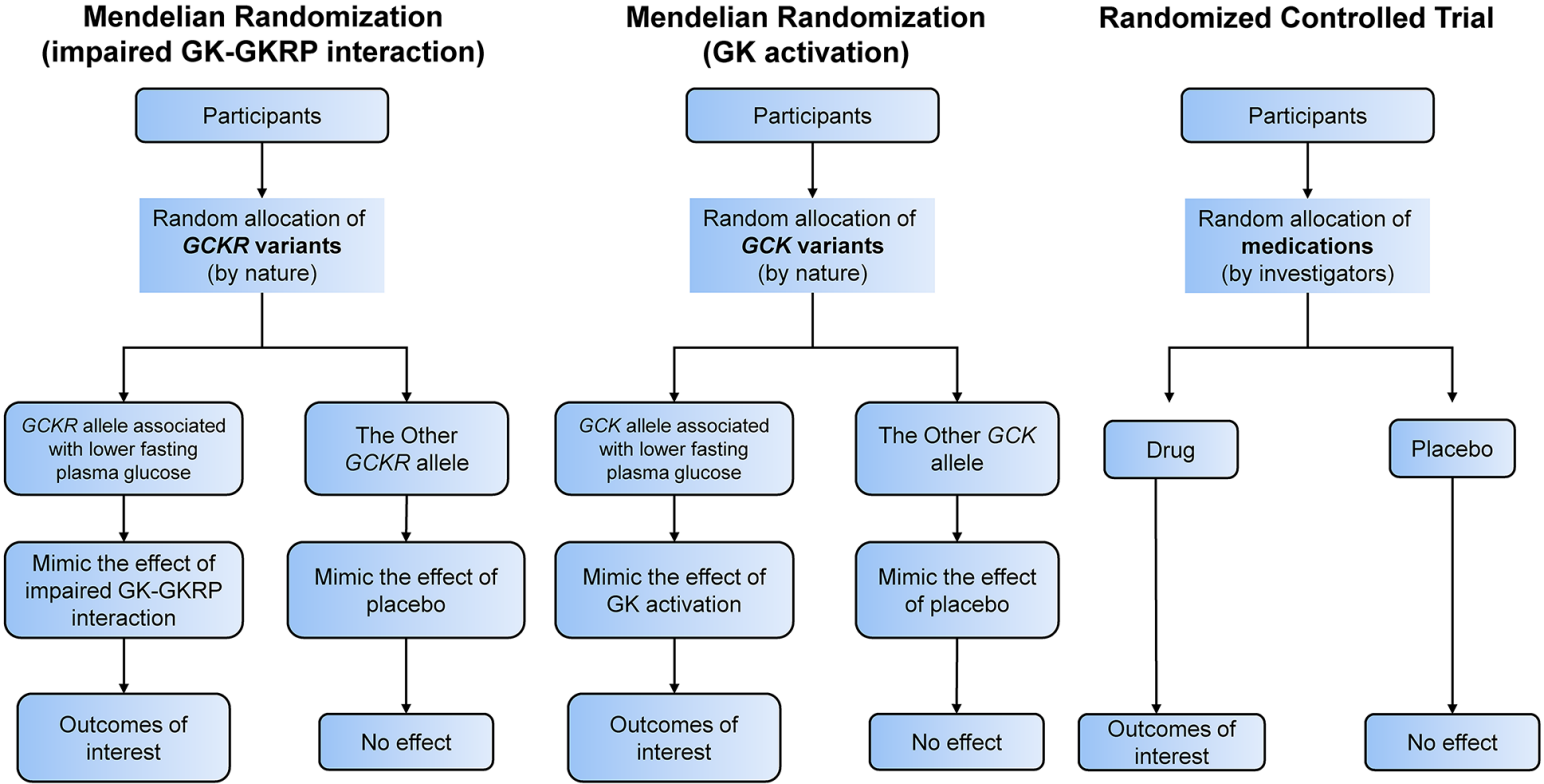
Conceptual framework of study design. GK, glucokinase; GKRP, glucokinase regulatory protein

We first conducted a two-sample MR analysis using publicly available summary statistics from genome-wide association studies (GWAS), followed by a one-sample MR validation using in-house data from the Hong Kong Diabetes Register (HKDR). The rise in plasma glucose is sensed by GK expressed in beta-cells in pancreatic islets. This is followed by release of insulin which promotes hepatic glycogen synthesis by GK in the liver. The hepatic activity of GK is regulated by GKRP which is exclusively expressed in the liver. Here, GK-GKRP is formed as a complex in the nucleus. Influx of glucose in the liver will trigger release of GK from the complex to the cytoplasm to promote glycogen synthesis. Impaired GK-GKRP interaction can lead to excessive GK activity with increased glucose influx into the liver causing low fasting plasma glucose (FPG) and if the capacity of glycogen synthesis is exceeded, de novo lipogenesis can follow. This provides the basis of utilizing *GCKR* single nucleotide polymorphisms (SNPs) to mimic the hepatic GK-GKRP disruption and *GCK* SNPs to mimic the GK activation in the pancreas and liver with low FPG*.* The low FPG level due to mimicked impaired GK-GKRP interaction or GK activation was regarded as the exposure. Lipid-related traits, MASLD and cardiovascular complications were the outcomes of interest. Figure 1 compares the conceptual framework of our analyses versus that of randomized controlled trial. All contributing studies in the MR analyses had received appropriate ethical approval and patient consent.

### Instrumental variables selection

We identified SNPs in *GCKR* gene (genomic position chromosome 2: 27719706–27746551 on build GRCh37.p13) associated (P ≤ 5 × 10^−8^) with FPG in the Meta-Analyses of Glucose and Insulin-related traits Consortium (MAGIC) [27] as proxies for pharmacological disruption of GK-GKRP interaction. We used linkage disequilibrium (LD) clumping algorithm in the PLINK (window size = 1000 KB, r^2^ threshold = 0.01, European LD reference panel from the 1000 Genomes Project) to filter and retain SNPs with the strongest significance in the MAGIC. For IVs selection, we limited the GWAS data from MAGIC to European population (N = 200,622) to avoid biases from population difference.

### Outcome data sources

In the two-sample MR study, we investigated the impact of genetically-proxied impaired GK-GKRP interaction on lipid-related traits including plasma triglyceride (TG), low-density lipoprotein cholesterol (LDL-C), high-density lipoprotein cholesterol (HDL-C) and apolipoprotein B (ApoB) levels as well as MASLD and cardiovascular diseases including CAD, peripheral arterial disease (PAD), stroke and HF, as defined by the respective Consortiums and the original studies. We employed incident T2D as positive control and insulin level as negative control given that GK-GKRP disruptors were designed to treat diabetes without influencing insulin secretion [17].

Additional file 1: Table S1 shows the sources of summary statistics from publicly available data used in our study: (1) TG (n = 441,016), LDL-C (n = 440,546), HDL-C (n = 403,943), ApoB (n = 439,214) levels from the UK BioBank [28]; (2) MASLD (reported as NAFLD in the original GWAS) from a meta-analysis published by Ghodsian et al. (8434 cases and 770,180 controls) [29]; (3) CAD from a meta-analysis of the CARDIOGRAMplusC4D Consortium and UK BioBank (122,733 cases and 424,528 controls) [30]; (4) PAD from the FinnGen Consortium (7098 cases and 206,541 controls); (5) stroke from the MEGASTROKE Consortium (40,585 cases and 406,111 controls) [31]; (6) HF from the Heart Failure Molecular Epidemiology for Therapeutic Targets (HERMES) Consortium (47,309 cases and 930,014 controls) [32]; (7) T2D from the 70KforT2D study (12,931 cases and 57,196 controls) [33] and (8) plasma insulin level from a GWAS published by Sun et al. (3301 samples) [34]. These summary statistics were all derived from subjects of European ancestry and the populations did not overlap with those in the MAGIC. Details of disease definitions and diagnoses are available in the respective Consortium websites or original publications and are briefly summarized in Additional file 1: Supplementary Method.

### One-sample MR validation

We performed a one-sample MR study of impaired GK-GKRP interaction in the HKDR for internal validation. The HKDR was established in 1995 as a quality improvement initiative at the Prince of Wales Hospital, the teaching hospital of the Chinese University of Hong Kong. The register consecutively enrolled patients referred to the Diabetes Mellitus and Endocrine Centre with documentation of detailed phenotypes at baseline and outcome data during follow-up. Once enrolled, the patients were monitored for the development of diabetic complications until their death. Using a unique identifier, clinical data on drug dispensing, laboratory results and hospitalization diagnosis were regularly retrieved from the electronic medical record (EMR) system of Hong Kong Hospital Authority. Patients provided written informed consent for allowing the anonymous use of their clinical data for the purpose of conducting clinical research. A portion of patients also consented to the collection of blood samples for future genetic and biomedical analyses. This project received ethical approval from the Clinical Research Ethics Committee of the Chinese University of Hong Kong. Details of the HKDR have been previously described [35] (CREC 2009-421 CREC 2019-080).

In the two-sample MR analyses, we only identified one *GCKR* SNP (rs1260326) as the IV, which was applied to the one-sample MR in a sub-cohort of the HKDR [36]. Patients with rs1260326 T allele were considered to have impaired GK-GKRP interaction because the T allele was associated with lower FPG in the MAGIC. The exposure was FPG-lowering secondary to impaired GK-GKRP interaction. The outcomes included lipid traits, prevalent MASLD, CAD, PAD, stroke and HF as defined in the HKDR. The definitions of these diseases were summarized in Additional file 1: Table S2.

### Evaluating causal effects of GK activation

To mimic GK activation, we selected SNPs located in *GCK* gene (genomic position chromosome 7: 44182812–44229038 on build GRCh37.p13) as IVs. These variants were associated (P ≤ 5 × 10^−8^) with FPG in the MAGIC and were not in LD (r^2^ = 0.01) with other variants. The outcomes were the same as those investigated for impaired GK-GKRP interaction.

### Colocalization analysis

We conducted colocalization analysis to assess the IV assumptions validity to mitigate the possibility that the exposure and outcome might be causally influenced by different variants which happen to be correlated with each other (in LD) [37]. We utilized the “coloc” package in R software to determine the probability of shared causal variants between exposure and outcomes which presented significant causality. The posterior probabilities (PP) were generated using approximate Bayes factor (ABF) computation with 5 exclusive hypotheses as following: (i) no genetic association for either trait (PPH0); (ii) a genetic association exists for only the first trait (PPH1); (iii) a genetic association exists for only the second trait (PPH2); (iv) both traits exhibit associations but with distinct causal variants (PPH3); (v) both traits exhibit associations and share a single causal variant (PPH4). We set the prior probability (p1) of the variant being associated with trait 1 to 1 × 10^−4^; the probability (p2) of the variant being associated with trait 2 to 1 × 10^−4^; and the probability (p12) of the variant being associated with both traits to 1 × 10^−5^. The exposure and the outcome were considered to have strong evidence of colocalization if PPH4 ≥ 0.8 and medium evidence if 0.6 ≤ PPH4 < 0.8. Colocalization analysis was carried out by generating ± 100 kb windows from the *GCKR* and *GCK* gene regions.

### Statistical analysis

We computed the *F*-statistic for IVs used in two-sample MR analyses to assess the robustness of instruments [38]. We defined the FPG-reducing allele as the effect allele of each SNP in the MAGIC, in line with the expected effects of impaired GK-GKRP interaction and direct GK activation. The genetic associations of IVs with the exposures and outcomes were then harmonized by aligning the effect alleles. The causal estimates of each SNP were calculated using Wald-ratio method [39]. The causal relationships were assessed by combining the Wald-ratio using the random-effects inverse variance-weighted (IVW) method if multiple SNPs were selected as IVs. Heterogeneity and horizontal pleiotropy for IVs which contained multiple SNPs were detected using Cochran’s Q statistic [40] and MR-Egger regression [41], respectively.

For the one-sample MR, the Wald-ratio method was used to calculate the causal estimates [39]. Firstly, the association of rs1260326 genotype with FPG was tested using multivariable linear regression to obtain the β-coefficient of exposure on IV. The associations of rs1260326 genotype with lipid traits and complications were assessed using multivariable linear or logistic regressions to obtain the β-coefficients of outcomes on IV. We only adjusted for covariates including age, sex and diabetes duration. Further adjustment was not implemented as it might bias estimates if the variable was on the causal pathway, or the adjustment induced collider bias [42, 43]. The Wald-ratio of one-sample MR was calculated using the formula: βiv-outcome/βiv-exposure, and the standard error (SE) was calculated via the delta method [44].

All analyses were performed using R 4.1.2 software with packages “TwoSampleMR” and “coloc”. A *P*-value of < 0.05 was deemed statistically significant.

## Results

### Two-sample MR for impaired GK-GKRP interaction

Only one SNP (Additional file 1: Table S3) in *GCKR* gene was identified from the MAGIC (European ancestry) as the IV (*F*-statistic = 275) to mimic impaired GK-GKRP interaction. Genetically-proxied impaired GK-GKRP interaction was associated with reduced risk of T2D (odds ratio [OR] 0.09 per 1 mmol/L lower FPG, 95% confidence interval [CI] 0.03–0.28, P = 2.53 × 10^−5^) and did not influence plasma insulin level (β − 1.43 per 1 mmol/L lower FPG, 95% CI − 3.19 to 0.33, *P* = 0.111) (Tables 1 and 2). These expected associations confirmed the validity of our IV. We found that genetically-proxied impaired GK-GKRP interaction significantly increased TG level (β 3.54 per 1 mmol/Llower FPG, 95% CI 3.26–3.82, P = 2.29 × 10^−132^), LDL-C level (β 1.23 per 1 mmol/L lower FPG, 95% CI 1.08–1.38, P = 5.13 × 10^−60^) and ApoB level (β 1.76 per 1 mmol/L lower FPG, 95% CI 1.61–1.90, P = 1.93 × 10^−121^), while decreased HDL-C level (β − 0.16 per 1 mmol/L lower FPG, 95% CI − 0.30 to − 0.03, *P* = 0.018) (Table 1).

**Table 1:** Causal associations of genetically-proxied impaired GK-GKRP interaction and GK activation with continuous outcomes in two-sample MR analyses

Exposure	Outcome	Beta (95% CI)	*P*
Genetically-proxied impaired GK-GKRP interaction (per 1 mmol/L lower FPG) instrumented by 1 *GCKR* SNP	Insulin	− 1.43 (− 3.19, 0.33)	0.111
	Triglycerides	3.66 (3.52, 3.80)	< 2.2 × 10^−308^
	LDL-C	1.23 (1.08, 1.38)	5.13 × 10^−60^
	HDL-C	− 0.16 (− 0.30, − 0.03)	0.018
	ApoB	1.76 (1.61, 1.90)	1.93 × 10^−121^
Genetically-proxied GK activation (per 1 mmol/L lower FPG) instrumented by 6 *GCK* SNPs	Insulin	0.84 (− 0.01, 1.69)	0.051
	Triglycerides	− 0.17 (− 0.31, − 0.02)	0.025
	LDL-C	− 0.00 (− 0.13, 0.12)	0.971
	HDL-C	0.09 (0.02, 0.16)	0.007
	ApoB	− 0.05 (− 0.19, 0.09)	0.483

Estimations were based on the Wald-ratio method or inverse variance-weighted method. CI, confidence interval; GK, glucokinase; GKRP, glucokinase regulatory protein; SNP, single nucleotide polymorphism; LDL-C, low-density lipoprotein cholesterol; HDL-C, high-density lipoprotein cholesterol; ApoB, apolipoprotein B

**Table 2:** Causal associations of genetically-proxied impaired GK-GKRP interaction and GK activation with binary outcomes in two-sample MR analyses

Exposure	Outcome	Case/control	OR (95% CI)	*P*	
Genetically-proxied impaired GK-GKRP interaction (per 1 mmol/L lower FPG) instrumented by 1 *GCKR* SNP	T2D	12,931/57,196	0.09 (0.03, 0.28)	2.53 × 10^−5^	
	MASLD	8434/770,180	14.6 (4.57, 46.4)	5.98 × 10^−6^	
	CAD	122,733/424,528	2.92 (1.78, 4.81)	2.44 × 10^−5^	
	HF	47,309/930,014	1.36 (0.78, 2.39)	0.283	
	PAD	7098/206,541	3.17 (0.83, 12.15)	0.091	
	Stroke	40,585/406,111	1.25 (0.63, 2.51)	0.522	
Genetically-proxied GK activation (per 1 mmol/L lower FPG) instrumented by 6 *GCK* SNPs	T2D	12,931/57,196	0.31 (0.17, 0.56)	9.88 × 10^−5^	P_heterogeneity_ = 0.626; P_pleiotropy_ = 0.940
	MASLD	8434/770,180	0.76 (0.42, 1.39)	0.374	
	CAD	122,733/424,528	0.69 (0.54, 0.88)	0.003	P_heterogeneity_ = 0.738; P_pleiotropy_ = 0.711
	HF	47,309/930,014	0.77 (0.58, 1.02)	0.071	
	PAD	7098/206,541	0.86 (0.42, 1.76)	0.681	
	Stroke	40,585/406,111	1.00 (0.70, 1.43)	0.995	

Estimations were based on the Wald-ratio method or inverse variance-weighted method. OR, odds ratio; CI, confidence interval; GK, glucokinase; GKRP, glucokinase regulatory protein; FPG, fasting plasma glucose; SNP, single nucleotide polymorphism; T2D, type 2 diabetes; MASLD, metabolic dysfunction-associated steatotic liver disease (reported as non-alcoholic fatty liver disease in the original study); CAD, coronary artery disease; HF, heart failure; PAD, peripheral arterial disease

Table 2 shows the causal relationships with binary outcomes of interest. Genetically-proxied impaired GK-GKRP interaction was associated with increased risk of MASLD (OR 14.6 per 1 mmol/L lower FPG, 95% CI 4.57–46.4, P = 5.98 × 10^−6^), as well as increased risk of CAD (OR 2.92 per 1 mmol/L lower FPG, 95% CI 1.78–4.81, P = 2.44 × 10^−5^). We did not observe any significant causal association between genetically-proxied impaired GK-GKRP interaction and risks of PAD (OR 3.17 per 1 mmol/L lower FPG, 95% CI 0.83–12.15, *P* = 0.091), stroke (OR 1.25 per 1 mmol/L lower FPG, 95% CI 0.63–2.51, *P* = 0.522), or HF (OR 1.36 per 1 mmol/L lower FPG, 95% CI 0.78–2.39, *P* = 0.283).

### One-sample MR validation

The SNP (*GCKR* rs1260326) identified in the two-sample MR analyses was utilized as the IV in our one-sample MR validation. In additional file 1, Table S4 shows the patients’ characteristics stratified by the number of *GCKR* rs1260326 effect allele (allele T). A total of 6072 patients were included in this one-sample MR analysis. FPG level was lower in patients with rs1260326 T allele, confirming the validity of IV selection. Plasma TG level and use of lipid-lowering drugs were higher in T allele carriers, consistent with the expected downstream effects of GK-GKRP disruption. The IV was associated with decreased FPG (β_IV-exposure_ − 0.22 mmol/L per T allele, SE 0.06, 95% CI − 0.10 to − 0.33, *P* = 0.0003) in an additive model. Each copy of T allele was also associated with higher level of TG (β_IV-outcome_ 0.15 mmol/L per T allele, SE 0.03, 95% CI 0.08–0.22, P = 1.56 × 10^−5^) (Additional file 1: Table S5). Other associations of IV with outcome were not significant (Additional file 1: Table S5).

The causal effects of genetically-proxied impaired GK-GKRP interaction on outcomes were estimated using the Wald-ratio method. Genetically-proxied impaired GK-GKRP interaction was causally associated with TG level (β_wald_ 0.69 mmol/L per 1 mmol/L lower FPG, 95% CI 0.20–1.18, *P* = 0.005). Though not significant, the causal effects on MASLD and cardiovascular outcomes also tended toward increased risk (Table 3).

**Table 3:** Causal associations of genetically-proxied impaired GK-GKRP interaction with outcomes in one-sample MR analyses using prospective data from the Hong Kong Diabetes Register

Exposure	Outcome	Sample size	Beta (95% CI)	*P*
Genetically-proxied impaired GK-GKRP interaction (per 1 mmol/L lower FPG instrumented by *GCKR* rs1260326)	Triglycerides	6043	0.69 (0.20, 1.18)	0.005
	LDLC	5783	− 0.14 (− 0.33, 0.04)	0.128
	HDLC	6009	− 0.04 (− 0.11, 0.02)	0.180

Exposure	Outcome	Event/total	OR (95% CI)	*P*
Genetically-proxied impaired GK-GKRP interaction (per 1 mmol/L lower FPG instrumented by *GCKR* rs1260326)	MASLD	48/6072	5.12 (0.66, 39.93)	0.119
	CAD	1407/6072	1.37 (0.89, 2.12)	0.150
	HF	883/6072	1.23 (0.75, 2.03)	0.420
	PAD	566/6072	1.18 (0.66, 2.11)	0.580
	Stroke	945/6072	1.39 (0.85, 2.30)	0.190

Estimations were based on the Wald-ratio method. OR, odds ratio; CI, confidence interval; GK, glucokinase; GKRP, glucokinase regulatory protein; FPG, fasting plasma glucose; LDL-C, low-density lipoprotein cholesterol; HDL-C, high-density lipoprotein cholesterol; MASLD, metabolic dysfunction-associated steatotic liver disease; CAD, coronary artery disease; HF, heart failure; PAD, peripheral arterial disease

### Causal effects of GK activation

Six *GCK* SNPs (Additional file 1: Table S6) associated with FPG were identified from the MAGIC as IVs (F-statistic = 171) to mimic GK activation. Genetically-proxied GK activation was associated with reduced risk of T2D (OR 0.31 per 1 mmol/L lower FPG, 95% CI 0.17–0.56, P = 9.88 × 10^−5^) and higher plasma insulin level (β 0.84 per 1 mmol/L lower FPG, 95% CI − 0.01 to 1.69, *P* = 0.051) (Tables 1 and 2). These expected associations confirmed the validity of the IV selection. Genetically-proxied GK activation was associated with decreased TG level (β − 0.17 mmol/L per 1 mmol/L lower FPG, 95% CI − 0.31 to − 0.02, *P* = 0.025) and increased HDL-C level (β 0.09 mmol/L per 1 mmol/L lower FPG, 95% CI 0.02–0.16, *P* = 0.007) (Table 1) but not with increased LDL-C and ApoB levels (Table 1). As for complications, genetically-proxied GK activation was associated with reduced risk of CAD (OR 0.69 per 1 mmol/L lower FPG, 95% CI 0.54–0.88, *P* = 0.003) and a tendency for reduced risk of HF (OR 0.77 per 1 mmol/L lower FPG, 95% CI 0.58–1.02, *P* = 0.071). Genetically-proxied GK activation was not causally associated with risks of MASLD (OR 0.76, 95% CI 0.42–1.39, *P* = 0.374), PAD (OR 0.86, 95% CI 0.42–1.76, *P* = 0.681) or stroke (OR 1.00, 95% CI 0.70–1.43, *P* = 0.995).

### Posterior probabilities of colocalization analysis

We carried out colocalization analysis to test potential confounding due to LD between SNPs linked to exposure and outcomes (Table 4). The posterior probabilities suggested that genetically-proxied impaired GK-GKRP interaction shared common causal variants with most outcomes (PPH4 = 0.991 for TG; PPH4 = 1.00 for ApoB; PPH4 = 1.00 for LDL-C; PPH4 = 0.985 for MASLD; PPH4 = 0.954 for CAD), providing strong evidence for colocalization. Genetically-proxied GK activation also shared common causal variant with CAD (PPH4 = 0.685), but distinct causal variants might exist between GK activation and TG (PPH3 = 0.886).

**Table 4:** Posterior probabilities of colocalization analysis in two-sample MR analyses

Exposure	Outcome	PPH0	PPH1	PPH2	PPH3	PPH4
Genetically-proxied impaired GK-GKRP interaction	Triglycerides	0.00E + 00	8.20E−304	4.73E−60	8.86E−03	9.91E−01
	ApoB	1.96E−174	4.08E−117	4.80E−61	1.11E−08	1.00E + 00
	LDL-C	3.56E−113	7.42E−56	4.80E−61	9.25E−08	1.00E + 00
	HDL-C	4.34E−58	9.05E−01	3.48E−59	7.25E−02	7.25E−02
	MASLD	2.27E−60	4.73E−03	5.39E−60	1.02E−02	9.85E−01
	CAD	1.76E−59	3.66E−02	5.14E−60	9.75E−03	9.54E−01
Genetically-proxied GK activation	Triglycerides	2.43E−179	5.01E−13	4.31E−167	8.86E−01	1.14E−01
	HDLC	3.54E−167	7.29E−01	1.36E−168	2.78E−02	2.44E−01
	CAD	1.44E−167	2.95E−01	9.88E−169	1.96E−02	6.85E−01

The population was restricted to European ancestry. GK, glucokinase; GKRP, glucokinase regulatory protein; PPH, posterior probability hypothesis; LDL-C, low-density lipoprotein cholesterol; HDL-C, high-density lipoprotein cholesterol; ApoB, apolipoprotein B. MASLD, metabolic dysfunction-associated steatotic liver disease (reported as non-alcoholic fatty liver disease in the original study); CAD, coronary artery disease

## Discussion

In this study, we utilized MR frameworks to evaluate the causal associations of impaired GK-GKRP interaction as well as GK activation with lipid traits and complications. We provided genetic evidence suggesting that impaired GK-GKRP interaction could worsen lipid profiles with increased risks of MASLD and CAD. In contrast, GK activation had minimal effects on lipid profiles with slightly lowered TG and increased HDL-C levels. GK activation also decreased the risk of CAD. These results suggested that disruption of GK-GKRP interaction, rather than GK activation per se, could worsen lipid profiles and increase risk of MASLD and cardiovascular complications.

In 2022, the first GKA (dorzagliatin) had been approved for treatment of T2D in China and so far, no data (more than 52 weeks) are available on its long-term effects. A previous MR study investigated the impact of GK activation (instrumented by *GCK* SNPs associated with HbA1c) on cardiovascular outcomes, suggesting its possible protective effects against CAD and HF [26]. However, it should be noted that some GKAs have been associated with dyslipidaemia, notably hypertriglyceridemia [16] although this was not reported with Dorzagliatin [23, 24]. This adverse effect is identical to the side effect of GK-GKRP disruptors. Since GKRP is exclusively expressed in the liver, it is reasonable to utilize *GCKR* SNPs to mimic impaired GK-GKRP interaction in hepatocytes where GK-GKRP disruption might lead to excessive glucose flux and de novo lipogenesis. Since some GKAs are known to interfere GK-GKRP interaction [13], our detailed MR analyses suggested that the risk of hypertriglyceridemia reported with some GKAs might be attributed to enhanced nuclear GK translocation to cytoplasm but not GK activation.

In the two-sample MR analysis, genetic variants located in *GCKR* gene associated with FPG level in the MAGIC were selected as IVs. Only one SNP, rs1260326, met the stringent selection criteria. This polymorphism (c.1337C > T; p.P446L) is a non-synonymous variant known to impair GK-GKRP interaction in the liver, with enhanced GK translocation from the GK-GKRP complex in the nucleus to the cytoplasm [45], making rs1260326 a reliable proxy. In this study, impaired GK-GKRP interaction instrumented by rs1260326 was associated with decreased risk of T2D without affecting plasma insulin level, further confirming its validity as an IV. Enhanced hepatic de novo lipogenesis due to GK-GKRP disruption may contribute to hepatic lipid accumulation, which plays an important role in the development of MASLD [22]. While cardiovascular diseases share some common cardiometabolic risk factors with MASLD, such as dyslipidaemia, through inflammation and insulin resistance, MASLD can independently promote atherosclerotic plaque formation and progression of cardiovascular disease [21, 46]. In keeping with the shift of glucose to lipid metabolism when the capacity of GK for hepatic glycogen synthesis is exceeded [15, 47], we found genetically-proxied impaired GK-GKRP interaction causally increased plasma TG, LDL-C, ApoB levels, decreased HDL-C level as well as increased risks of MASLD and CAD. Causal associations for HF, PAD and stroke tended toward increased risk, albeit not significant. We validated the effect direction derived from the two-sample MR framework by a one-sample MR analysis in a local, prospective cohort HKDR. The causal estimates in one-sample MR were directionally consistent with those observed in the two-sample MR, supporting the causal impact of GK-GKRP disruption on abnormal lipid metabolism, MASLD and CAD. Colocalization analysis has been demonstrated as an effective method for uncovering the potential pleiotropic effects of specific loci on multiple traits [37]. The PPH4 values derived from our colocalization analyses also suggested that genetically-proxied impaired GK-GKRP interaction shared common causal variants with most outcomes which showed significant association in the MR analyses, proving the absence of pleiotropic effects in IV selection.

We also evaluated impacts of GK activation on these outcomes using *GCK* SNPs associated with FPG as IVs. The results were overall consistent with our previous analysis using *GCK* SNPs associated with HbA1c as IVs [26]. In contrast to the impaired GK-GKRP interaction, GK activation did not influence the lipid traits. The slightly decreased TG level might be due to increased insulin secretion with improved clearance of TG from the circulation [48]. Taken together, we inferred that the hypertriglyceridemia risk during GKA development might be better explained by increased cytoplasm GK level in hepatocytes due to enhanced GK translocation, rather than due to up-regulation of hepatic GK activity via conformational change. In mice models fed with normal diet, GK overexpression in the liver lowered blood glucose accompanied by increased hepatic lipogenesis and circulating lipids [49]. In human liver biopsies, increased *GCK* mRNA expression was associated with increased mRNA expression of hepatic lipogenic enzymes and liver fat [50]. On the other hand, activating *GCK* mutations in mice and human were associated with increased risk of hypoglycemia but not altered circulating lipid profiles [51–53].

For decades, GKAs and GK-GKRP disruptors have been investigated as novel OADs. In this study, utilizing MR framework, we have dissected the different effects of GK activation and GK-GKRP disruption on lipid levels. Whilst both could lower PG, the hyperlipidemic side effects of GK-GKRP disruption might outweigh its glucose-lowering effects with possible adverse long-term outcomes including MASLD and cardiovascular disease. This inference was supported by previous findings that *GCKR* rs780094 (in high linkage disequilibrium with rs1260326) was associated with a modest decrease in FPG (1.9%) but a proportionately larger increase in plasma TG level (13%) [54]. These adverse effects of GK-GKRP disruption may be particularly relevant to patients with diabetes, who are prone to develop dyslipidemia, notably high TG and low HDL-C, due to insufficient insulin action [55].

We acknowledge certain limitations in our study and the MR results should be interpreted with caution. For MR study, there are three core assumptions: (1) the IV should be associated with the exposure; (2) the IV should not be associated with confounders of the exposure-outcome association; (3) the IV should not directly affect the outcome except through its influence on the exposure. However, the latter two core assumptions can never be entirely proven. Our study is a drug-target MR framework. By employing IVs in specific genes (instead of the whole genome) where their functions and relationships with the exposure are well, we can reduce the risk of confounding and pleiotropy bias. 

We acknowledge that *GCKR* rs1260326 is a pleiotropic SNP associated with multiple traits and dozens of metabolites [19]. The latter are likely due to the downstream effects of impaired GK-GKRP interaction. Although such ‘vertical pleiotropy’ does not interfere with the interpretation of our MR analysis, given its multifaceted associations, it remains possible that rs1260326 might be related to some unknown traits independent of the causal pathway (horizontal pleiotropy). 

In addition, most MR studies used GWAS data collected in mid-life, long after the random allocation of genetic variants at conception. Thus, we were unable to account for potential selection bias stemming from competing risks before enrolment, for example, for diseases that typically occur at younger ages which share the same risk factors. Besides, the reported effect sizes cannot be directly utilized to extrapolate the clinical effects of GKA or GK-GKRP disruptor treatment which will depend on the period of treatment exposure. Most randomized controlled trials only investigated the short-term effects of pharmacological treatment as opposed to the impacts of lifelong exposures estimated by MR [56]. We only validated our findings in a local cohort and larger validation studies in other ethnic groups are warranted*.* Moreover, our study is a biomarker (FPG) weighted drug MR analysis. Using a protein expression weighted drug MR framework to assess whether perturbation of the hepatic GK protein level could also influence the outcomes. would further strengthen our findings [57]. However, high-quality data for GK protein expression are not currently available.

## Conclusion

Our genetic evidence suggested that impaired interaction of GK-GKRP complex may increase the risk of liver and cardiovascular diseases, whereas direct GK activation can protect from cardiovascular diseases. Hypertriglyceridemia observed in previous clinical trials with some GKAs are most probably due to unexpected impaired GK-GKRP interaction during GK activation. Future development of GKA needs to avoid interfering GK-GKRP interaction. These findings would need to be confirmed by definitive clinical trials.

### Supplementary Information

Additional file 1.

## Data Availability

The summary statistics used in two-sample MR analyses are publicly available and can be accessed and downloaded through websites listed in Table S1. Due to local laws and regulations, the individual data of the HKDR cannot be shared. Further enquiries can be made to Dr. Juliana CN Chan at jchan@cuhk.edu.hk.

## Abbreviations

ApoB: Apolipoprotein B
CAD: Coronary artery disease
CI: Confidence interval
FPG: Fasting plasma glucose
GK: Glucokinase
GKA: Glucokinase activator
GKRP: Glucokinase regulatory protein
GWAS: Genome-wide association studies
HDL-C: High-density lipoprotein cholesterol
HF: Heart failure
HKDR: Hong Kong Diabetes Register
IV: Instrumental variable
IVW: Inverse variance-weighted
LDL-C: Low-density lipoprotein cholesterol
MAGIC: Meta-analyses of glucose and insulin-related traits consortium
MALSD: Metabolic dysfunction-associated steatotic liver disease
MODY: Maturity-onset diabetes of the young
MR: Mendelian randomization
NAFLD: Non-alcoholic fatty liver disease
OAD: Oral antidiabetic drug
OR: Odds ratio
PAD: Peripheral arterial disease
PHHI: Persistent hyperinsulinemic hypoglycemia of infancy
SNP: Single nucleotide polymorphisms
TG: Triglycerides
T2D: Type 2 diabetes
