# Supplementary material for: Impaired GK-GKRP interaction rather than direct GK activation worsens lipid profiles and contributes to long-term complications: a Mendelian randomization study
Source: Cardiovasc Diabetol. 2024 Jun 29;23:228. doi: 10.1186/s12933-024-02321-z (PMC11218184; doi:10.1186/s12933-024-02321-z)
Supplement: Supplementary file 1 — Additional file 1. [file 12933_2024_2321_MOESM1_ESM.docx]

Table S1. Information of included summary statistics in two-sample Mendelian randomization

| Trait or disease | Consortium or cohort study | Participants | Web source and publication |
| --- | --- | --- | --- |
| Fasting plasma glucose | Meta-Analyses of Glucose and Insulin-related traits Consortium (MAGIC) | 200,622 European-descent individuals | https://magicinvestigators.org/downloads/  PMID: 34059833 |
| Type 2 diabetes | 70KforT2D study | 12,931cases and 57,196 controls of European ancestry | <https://www.ebi.ac.uk/gwas/studies/GCST005413>  PMID: 29358691 |
| Plasma insulin | INTERVAL study | 3,301 European-descent individuals | <https://www.ebi.ac.uk/gwas/studies/GCST005806>  PMID: 29875488 |
| Triglycerides level | UK BioBank | 441,016 European-descent individuals | https://gwas.mrcieu.ac.uk/datasets/ieu-b-111/  PMID: 32203549 |
| Low-density lipoprotein cholesterol level | UK BioBank | 440,546 European-descent individuals | https://gwas.mrcieu.ac.uk/datasets/ieu-b-110/  PMID: 32203549 |
| High-density lipoprotein cholesterol level | UK BioBank | 403,943 European-descent individuals | https://gwas.mrcieu.ac.uk/datasets/ieu-b-109/  PMID: 32203549 |
| Apolipoprotein B level | UK BioBank | 439,214 European-descent individuals | https://gwas.mrcieu.ac.uk/datasets/ieu-b-108/  PMID: 32203549 |
| Non-alcoholic fatty liver disease (metabolic dysfunction-associated steatotic liver disease) | Meta-analyses of the eMERGE network, the UK Biobank, the Estonian Biobank and FinnGen | 8,434 cases and 770,180 controls of European ancestry | https://www.ebi.ac.uk/gwas/studies/GCST90091033  PMID: 34841290 |
| Coronary artery disease | Meta analysis of CARDIOGRAMplusC4D Consortium and UK BioBank | 122,733 cases and 424,528 controls of European ancestry | https://www.ebi.ac.uk/gwas/studies/GCST005195  PMID: 29212778 |
| Peripheral arterial disease | FinnGen Consortium | 7,098 cases and 206,541 controls of European ancestry | <https://www.finngen.fi/en>  (Data freeze 5) |
| Stroke | MEGASTROKE consortium | 40,585 cases and 406,111 controls of European ancestry | <https://www.megastroke.org/>  PMID: 29531354 |
| Heart failure | Heart Failure Molecular Epidemiology for Therapeutic Targets (HERMES) Consortium | 47,309 cases and 930,014 controls of European ancestry | <https://www.hermesconsortium.org/>  PMID: 31919418 |

Table S2. Definitions of outcomes in the Hong Kong Diabetes Register

| Outcome | Definition |
| --- | --- |
| Metabolic dysfunction-associated steatotic liver disease | Diagnosis code 571.8 |
| Coronary artery disease | Acute myocardial infarction (diagnosis code 410), ischemic heart disease (diagnosis code 411-414), coronary revascularization (procedure code 36), or percutaneous transluminal coronary angioplasty or coronary atherectomy (procedure code 00.66) |
| Peripheral arterial disease | Diabetes with peripheral circulatory disorders (diagnosis code 250.7), gangrene (diagnosis code 785.4), angiopathy in diseases classified elsewhere (diagnosis code 443.81), PAD unspecified (diagnosis code 443.9), other peripheral vascular shunt or bypass (procedure code 39.29), insertion of non-drug-eluting peripheral vessel stents (procedure code 39.90), or amputation of lower limb (procedure code 84.1) without a traumatic amputation (diagnosis code 895-897) |
| Stroke | Subarachnoid (diagnosis code 430) and intracerebral (diagnosis code 431) hemorrhage, other and unspecified intracranial hemorrhage (diagnosis code 432), occlusion and stenosis of precerebral (diagnosis code 433) and cerebral (diagnosis code 434) arteries, or acute but ill-defined cerebrovascular disease (diagnosis code 436) |
| Heart failure | Diagnosis code 428 |

All diagnosis and procedure codes were based on the International Classification of Disease-9th revision

Table S3. Instrumental variables from Meta-Analyses of Glucose and Insulin-related traits Consortium (MAGIC) for impaired GK-GKRP interaction

| SNP | Position | Effect allele | Other allele | EAF | Effect size (FPG) | Standard error | P |
| --- | --- | --- | --- | --- | --- | --- | --- |
| rs1260326 | 2: 27730940 | T | C | 0.413 | -0.0282 | 0.0017 | 4.48E-65 |

SNP, single nucleotide polymorphism; EAF, effect allele frequency; FPG, fasting plasma glucose

Table S4. Patients’ characteristics stratified by number of *GCKR* rs1260326 effect allele in the Hong Kong Diabetes Register

|  | *GCKR* rs1260326 effect allele | | |  |
| --- | --- | --- | --- | --- |
|  | 0 | 1 | 2 | P |
| Patients (n) | 1814 (29.9%) | 2997 (49.4%) | 1261 (20.8%) |  |
| Age (years) | 57.27 (13.28) | 57.50 (13.29) | 57.35 (13.15) | 0.829 |
| Female (n) | 965 (53.2%) | 1649 (55.0%) | 695 (55.1%) | 0.414 |
| Diabetes duration (years) | 6.00 (2.00-11.00) | 6.00 (2.00-11.00) | 6.00 (2.00-11.00) | 0.952 |
| Active smokers (n) | 248 (13.7%) | 398 (13.3%) | 137 (10.9%) | 0.050 |
| Active alcohol drinkers (n) | 179 (9.9) | 260 (8.7) | 95 (7.6) | 0.077 |
| Body mass index (kg/m^2^) | 25.18 (3.95) | 25.19 (4.01) | 25.22 (3.99) | 0.950 |
| Systolic blood pressure (mmHg) | 134.84 (20.79) | 135.62 (21.02) | 136.21 (20.22) | 0.183 |
| Diastolic blood pressure (mmHg) | 75.47 (10.77) | 75.51 (10.99) | 76.10 (11.34) | 0.225 |
| Fasting plasma glucose (mmol/L) | 8.81 (3.46) | 8.67 (3.36) | 8.35 (2.96) | 0.001 |
| HbA_1c_ (%) | 7.69 (1.82) | 7.67 (1.81) | 7.57 (1.67) | 0.188 |
| Total cholesterol (mmol/L) | 5.19 (1.14) | 5.26 (1.17) | 5.24 (1.17) | 0.163 |
| Triglycerides (mmol/L) | 1.28 (0.90-1.92) | 1.40 (0.99-2.09) | 1.51 (1.02-2.30) | <0.001 |
| LDL-C (mmol/L) | 3.13 (1.01) | 3.13 (0.98) | 3.07 (1.02) | 0.119 |
| HDL-C (mmol/L) | 1.32 (0.38) | 1.31 (0.37) | 1.30 (0.35) | 0.467 |
| eGFR (mL/min/1.73m^2^) | 83.32 (63.61-98.82) | 83.66 (63.11-99.78) | 84.11 (62.73-99.15) | 0.980 |
| Use of anti-hypertensive drugs (n) | 807 (44.5%) | 1408 (47.0%) | 604 (47.9%) | 0.122 |
| Use of lipid-lowering drugs (n) | 315 (17.4%) | 507 (16.9%) | 284 (22.5%) | <0.001 |
| Use of oral glucose-lowering drugs (n) | 1218 (67.1%) | 1960 (65.4%) | 834 (66.1%) | 0.463 |
| Use of insulin (n) | 318 (17.5%) | 535 (17.9%) | 228 (18.1%) | 0.922 |

Means (standard deviation), medians (interquartile range) or number (percentage) of patient’s characteristics are presented. One-way analysis of variance or Kruskal-Wallis test were used for continuous variables. Chi-squared test was used for categorical variables. LDL-C, low-density lipoprotein cholesterol; HDL-C, high-density lipoprotein cholesterol; eGFR, estimated glomerular filtration rate.

Table S5. Associations between IV and outcomes in the Hong Kong Diabetes Register

| IV | Outcome | Event/total | Beta (SE) | Beta_95%CI | OR (95%CI) | P |
| --- | --- | --- | --- | --- | --- | --- |
| GCKR rs1260326 T allele | Triglycerides | 6043/6043 | 0.15 (0.03) | (0.08, 0.22) | NA | 1.56×10^−5^ |
|  | LDLC | 5783/5783 | -0.03 (0.02) | (-0.07, 0.01) | NA | 0.094 |
|  | HDLC | 6009/6009 | -0.01 (0.01) | (-0.02, 0) | NA | 0.149 |
|  | MASLD | 48/6072 | 0.35 (0.21) | (-0.05, 0.76) | 5.12 (0.66, 39.93) | 0.084 |
|  | CAD | 1407/6072 | 0.07 (0.04) | (-0.02, 0.16) | 1.37 (0.89, 2.12) | 0.117 |
|  | HF | 883/6072 | 0.04 (0.05) | (-0.06, 0.15) | 1.23 (0.75, 2.03) | 0.408 |
|  | PAD | 566/6072 | 0.04 (0.06) | (-0.09, 0.16) | 1.18 (0.66, 2.11) | 0.576 |
|  | Stroke | 945/6072 | 0.07 (0.05) | (-0.03, 0.17) | 1.39 (0.85, 2.30) | 0.160 |
|  |  |  |  |  |  |  |

All regressions were adjusted for age, sex, and diabetes duration. IV, instrumental variables; SE, standard error; OR, odds ratio; CI, confidence interval; LDL-C, low-density lipoprotein cholesterol; HDL-C, high-density lipoprotein cholesterol; MASLD, metabolic dysfunction-associated steatotic liver disease; CAD, coronary artery disease; HF, heart failure; PAD, peripheral arterial disease.

Table S6. Instrumental variables from Meta-Analyses of Glucose and Insulin-related traits Consortium (MAGIC) for GK activation

| SNP | Position | Effect allele | Other allele | EAF | Effect size (FPG) | Standard error | P |
| --- | --- | --- | --- | --- | --- | --- | --- |
| rs2908277 | 7:44183433 | A | G | 0.117 | 0.0296 | 0.0027 | 2.20E-30 |
| rs145391098 | 7:44191553 | A | G | 0.038 | -0.0336 | 0.0061 | 3.44E-09 |
| rs17832252 | 7:44200099 | T | G | 0.133 | -0.0157 | 0.0024 | 1.45E-10 |
| rs11766576 | 7:44220919 | A | C | 0.04 | -0.0432 | 0.0082 | 2.88E-08 |
| rs730497 | 7:44223721 | A | G | 0.179 | 0.0612 | 0.0022 | 1.88E-165 |
| rs77020674 | 7:44227979 | A | C | 0.034 | -0.0286 | 0.0051 | 4.19E-08 |

SNP, single nucleotide polymorphism; EAF, effect allele frequency; FPG, fasting plasma glucose

**Supplementary Method. Brief summary of disease definitions in two-sample MR analyses**

**Type 2 diabetes**

(https://www.ebi.ac.uk/gwas/studies/GCST005413, PMID: 29358691)

Cases from following cohorts:

| **Cohort** | **Definition** |
| --- | --- |
| *NuGENE NORTHWESTERN* | Patients with T2D diagnosis based on ICD9 code (excluding those with ketoacidosis codes).  Patients with HbA1c lab value ≥ 6.5%, fasting glucose > 125 mg/dl or random glucose > 200 mg/dl AND prescribed one of the antidiabetic medications. |
| *FUSION* | WHO 1999 criteria of fasting plasma glucose ≥ 7.0 mmol/l or 2-h plasma glucose ≥ 11.1 mmol/l, by report of diabetes medication use, or based on medical record review. |
| *GENEVA NHS/HPFS* | One or more classic symptoms plus fasting plasma glucose ≥ 140 mg/dl (7.8 mmol/L) and/or random plasma glucose ≥ 200 mg/dl (11.1 mmol/L) and/or plasma glucose 2 hours after an oral glucose tolerance test ≥ 200 mg/dl; or at least two elevated plasma glucose levels (as described above) on different occasions in the absence of symptoms; or treatment with hypoglycaemic medication (insulin or oral hypoglycaemic agent). |
| *WTCCC* | Either current prescribed treatment with sulphonylureas, biguanides, other oral agents and/or insulin or, in the case of individuals treated with diet alone, historical or contemporary laboratory evidence of hyperglycaemia (as defined by the World Health Organization). Other forms of diabetes were excluded by standard clinical criteria. |
| *GERA* | At least two diagnoses within this disease category that had to be recorded on separate days. ICD9-CM diagnoses used for the T2D category were: 250.00; 250.02; 250.10; 250.12; 250.20; 250.22; 250.30; 250.32; 250.40; 250.42; 250.50; 250.52; 250.60; 250.62; 250.70; 250.72; 250.80; 250.82; 250.90; 250.92. |

**Non-alcoholic fatty liver disease (metabolic dysfunction-associated steatotic liver disease)**

(https://www.ebi.ac.uk/gwas/studies/GCST90091033, PMID: 34841290)

Cases from following cohorts:

| **Cohort** | **Definition** |
| --- | --- |
| *eMERGE* *network* | ICD9: 571.5, ICD9: 571.8, ICD9: 571.9, ICD10: K75.81, ICD10: K76.0 and ICD10: K76.9 |
| *UK Biobank* | ICD10: K74.0 and K74.2 (hepatic fibrosis), K75.8 (NASH), K76.0 (NAFLD) and ICD10: K76.9 (other specified diseases of the liver) |
| *Estonian Biobank* | ICD10: K74.0 and K74.2 (hepatic fibrosis), K75.8 (NASH), K76.0 (NAFLD) and ICD10: K76.9 (other specified diseases of the liver) |
| *FinnGen* | ICD10: K76.0. Data freeze 4. |

**Coronary artery disease**

(https://www.ebi.ac.uk/gwas/studies/GCST005195, PMID: 29212778)

Cases from UK biobank:

- ICD 10 codes: I21-I25 covering ischemic heart diseases
- Office of Population Censuses and Surveys Classification of Interventions and Procedures version 4 (OPCS-4) codes: K40-K46, K49, K50 and K75 which includes replacement, transluminal balloon angioplasty, and other therapeutic transluminal operations on coronary artery and percutaneous transluminal balloon angioplasty and insertion of stent into coronary artery
- Self-reported CAD: heart attack/myocardial infarction, coronary angioplasty +/- stent, cabg and triple heart bypass

Cases from CARDIoGRAM Consortium:

| **Cohort** | **Definition** |
| --- | --- |
| *ADVANCE* | Clinical non-fatal CAD (men ≤45 yrs, women ≤55 yrs) including AMI (enzymes), typical angina with ≥1 artery with >50% stenosis, positive non-invasive test, or PCI or CABG |
| *CADomics* | CAD: >50% stenosis in 1 major coronary artery and/or MI based on ECG and enzymes |
| *CHARGE* | CHD: definite or probable MI, PTCA or CABG, or ECG MI |
| *deCODE* CAD | MI: MONICA criteria (<75 yrs) or discharge diagnosis of MI; CAD: PCI or participation in CVD genetics program with self-report of CABG or PCI, or discharge diagnosis of angina pectoris, MI or chronic ischaemic heart disease |
| *GERMIFS* *I* | MI (<65 yrs) with >1 1st degree sibling with severe CAD (PTCA; MI; CABG) |
| *GERMIFS* *II* | MI (<60 yrs); 59.4% with family history of CAD |
| *GERMIFS* *III (KORA)* | MI (<60 yrs); MONICA criteria |
| *LURIC/ AtheroRemo 1* | Symptoms of angina pectoris, NSTEMI, STEMI, or >50% coronary stenosis |
| *LURIC/ AtheroRemo 2* | Symptoms of angina pectoris, NSTEMI, STEMI, or >50% coronary stenosis |
| *MedStar* | Angiography (≥1 coronary vessel with >50% stenosis); ≤55 for males and ≤60 for females. |
| *MIGen* | MI (men <50 yrs / women <60 yrs) |
| *OHGS1* | Angiographic (>50% stenosis) |
| *PennCATH* | Angiography (≥1 coronary vessel with >50% stenosis); ≤60 for males and ≤65 for females. |
| *WTCCC* | Validated MI, CABG, PTCA or angina with positive non-invasive testing <66 yrs |

**Peripheral artery disease**

(FinnGen Consortium data freeze 5, https://www.finngen.fi/en/researchers/clinical-endpoints)

PAD cases are defined as ICD 10th codes E105, E115, E125, E135, E145, I702 and I739; ICD 9th codes 4402 and 4439; ICD 8th codes 25006, 4402 and 4439.

**Stroke**

(MEGASTROKE consortium, https://www.megastroke.org/, PMID: 29531354)

Cases from following cohorts:

| **Cohort** | **Definition** |
| --- | --- |
| *ASGC* | Stroke was defined by WHO criteria as a sudden focal neurologic deficit of vascular origin, lasting more than 24 hours and confirmed by imaging such as computerised tomography (CT) and/or magnetic resonance imaging (MRI) brain scan. Other investigative tests such as electrocardiogram, carotid doppler and transoesophageal echocardiogram were conducted to define IS mechanism as clinically appropriate. |
| *BRAINS* | Diagnosis of stroke was confirmed using positive imaging (MRI or CT) and ischemic stroke subtypes were assigned using TOAST criteria, based on clinical, imaging and risk factor data. |
| *GEOS* | Cases were identified through discharge surveillance from 59 participating hospitals and direct physician referral from a defined geographic region. |
| *HPS* | Individuals entering HPS with a clinical diagnosis of ischemic stroke were used as cases in the METASTROKE study. |
| *ISGS* | All recruits were extensively clinically phenotyped and have imagingconfirmed ischemic stroke using either CT or MRI brain scans. Probands are adult men and women over the age of 18 years diagnosed with ischemic stroke confirmed by a study neurologist on the basis of history, physical examination and CT or MR imaging of the brain who also have a history of at least one living sibling with a history of stroke. |
| *MGH-GASROS* | Ischemic stroke was defined as either (1) a radiographically proven (head CT or MRI) infarct associated with the appropriate clinical stroke syndrome, or (2) a fixed neurological deficit persisting more than 24 hours, consistent with a vascular pattern of involvement and without radiographic evidence of demyelinating disease, or other non-vascular structural disease. |
| *Milano* | Ischemic stroke cases, first ever or recurrent, confirmed on brain imaging, were selected for this study. All cases were of selfreported Caucasian ancestry and had clinically relevant diagnostic workup performed. All cases were phenotyped by an experienced stroke neurologist according to TOAST criteria, based on relevant clinical imaging and available information on cardiovascular risk factors. |
| *WTCCC2* | All cases were of self-reported Caucasian ancestry. Ischemic stroke subtypes were determined according to TOAST criteria based on relevant clinical imaging and available information on cardiovascular risk factors. |
| *VISP* | Nondisabling cerebral infarction was defined as an ischemic brain infarction not due to embolism from a cardiac source, characterized by the sudden onset of a neurological deficit. |
| *WHI* | Stroke diagnosis requiring and/or occurring during hospitalization was based on rapid onset of a neurological deficit attributable to an obstruction or rupture of an arterial vessel system. Hospitalized incident stroke events were identified by semiannual questionnaires and adjudicated following medical record review, which occurred both locally and centrally. |
| *BASICMAR* | Ischemic stroke etiologic subtypes were classified according to TOAST criteria. |
| *GRAZ* | Ischemic stroke was defined as an episode of focal neurological deficits with acute onset and lasting > 24 hours. |
| *KRAKOW* | All cases were phenotyped independently by two experienced stroke neurologists with review of original imaging. Cases were subsequently classified additionally using the CCS system. |
| *LSGS* | Cases of European descent with cerebral ischemia, defined as a clinical stroke with imaging confirmation or a TIA with a new ischemic lesion on diffusion-weighted imaging. |
| *LSR* | Stroke was defined using the WHO criteria.12 Subjects aged 18 years or older with stroke caused by cerebral infarct, intracerebral hemorrhage or subarachnoid hemorrhage are included. |
| *MCISS* | All subjects with clinical suspicion of a stroke were admitted through the emergency room to a dedicated stroke unit supervised by a vascular neurologist. |
| *MIAMISR* | an ongoing prospective hospital registry of consecutive patients subjects with prevalent stroke (ischemic and hemorrhagic) and TIA with available neuroimaging (CT or MRI) who provide informed consent. |
| *NHS* | prospectively identified incident strokes and confirmed ischemic stroke cases by medical record review. Clinical symptoms consistent with stroke and exclusion of alternate etiologies were required for classification of stroke. |
| *NOMAS* | First-ever ischemic stroke cases were identified for the casecontrol study by screening of patient admissions, discharge codes, and referrals for neuroimaging at 15 acute care hospitals in the defined study area and multiple approaches to monitor for non-hospitalized cases. Incident ischemic stroke cases were identified from the prospective cohort study through follow-up visits and scheduled telephone contacts. |
| *REGARDS* | A symptom-based approach, independent of neuroimaging outcome, is used to confirm events using the WHO definition of stroke. |
| *SPS3* | Principal eligibility criteria include man or woman at least 30 years of age with clinical evidence of small subcortical stroke and brain MRI evidence of small subcortical infarct. |
| *WUSTL* | Subjects were retained in the study if their discharge diagnosis was ischemic stroke (without requirement for the stroke to be visualized on CT or MRI). |
| *AGES* | Incident stroke cases were ascertained from multiple sources including hospital, general practice, nursing home records and death certificates. All possible cases were adjudicated with standard TOAST criteria by two Neurologists and a Neuroradiologist with expertise in evaluating stroke cases for epidemiologic studies. |
| *CHS– European Ancestry* | Stroke definitions were derived from the criteria used for the Systolic Hypertension in the Elderly Program (SHEP). |
| *FHS* | Incident strokes have been identified since 1948 through this ongoing system of FHS clinic and local hospital surveillance; they include review of medical records and collaboration with local general practitioners, emergency rooms and imaging facilities. |
| *FINRISK* | ICD-codes: I63; not I63.6, I64 (ICD-10) / 4330A, 4331A, 4339A, 4340A, 4341A, 4349A, 436 (ICD-9) / 433, 434, 436 (ICD-8) for Ischemic stroke excluding any hemorrhagic strokes, and I60-I61,I63-I64 (not I63.6) (ICD-10) / 430, 431, 4330A, 4331A, 4339A, 4340A, 4341A, 4349A, 436 (ICD-9) / 430, 431 (except 431.01, 431.91), 433, 434, 436 (ICD-8) for allstroke including SAH. ICD-8 codes 430, 431 (excluding codes 431.01, 431.91 of the Finnish adaptation of ICD-8*), 432, 433, 434 or with ICD-9 codes 430, 431, 433 (excluding codes 4330X, 4331X, 4339X of the Finnish adaptation of ICD-9*), 434 (excluding code 4349X of the Finnish adaptation of ICD-9*), 436, 437, 438 or with ICD-10 codes I60, I61, I63 (excluding I63.6), I64 orI69 |
| *Health ABC* | Participants were screened for stroke events every 6 months alternating between semi-annual phone interviews and annual clinical visits. Any self-reported hospitalization for stroke led to medical record abstraction and verification by a Health ABC Disease Adjudicator at each site. |
| *Rotterdam* | For suspected stroke and TIA events, both fatal and non-fatal, additional information (including neuroimaging) was obtained from general practitioner’ and hospital records and research physicians discussed available information with an experienced stroke neurologist to verify all diagnoses and to subclassify the strokes. |
| *SHIP* | For in- and outpatient data any stroke was defined as cases with a coded ICD I61, I63, I64, I69.1, I69.3, I69.4 diagnosis. For ischemic stroke we included all cases with I63.x codes based on in- and outpatient data. |
| *WGHS* | A confirmed stroke was defined as a new neurologic deficit of sudden onset that persisted for >24 h. Clinical information as well as computed tomographic scans or MRI were used to distinguish hemorrhagic from ischemic events. |
| *MESA* | To verify self-reported diagnoses, information was collected from death certificates and medical records for all hospitalizations and outpatient cardiovascular diagnoses, using ICD-9 and ICD-10 codes. |
| *TWINGENE* | For stroke, the following ICD codes were used: ICD-8 codes 430–436, ICD-9 codes 430–436 and ICD-10 codes I60-I64 and G45.52 Further classification into stroke subtypes was done using ICD-8 codes 432-434, ICD-9 codes 433-434, and ICD-10 code I63 for ischemic stroke, and ICD-8 codes 430-431, ICD-9 codes 430-432, and ICD-10 codes I60-I62 for hemorrhagic stroke. |
| *ULSAM* | ICD-8 codes 430-431 and 433-434, ICD-9 codes 430-432, 434 or ICD-10 codes I60-I64. |
| *3C-Study* | Stroke was confirmed if the participant had a new focal neurological deficit of sudden onset attributable to a cerebrovascular event that persisted for more than 24 hours. |
| *EPIC* | Stroke events were defined by ICD10 codes as follows: Ischemic I63, Haemorrhagic I61, SAH I60, Unclassified I64, Other CRBV I62, I65- I69, F01. |
| AIDHS/SDS | Diagnosis of ischemic stroke was established based on either evidence of an infarction in neuroimaging (CT/MRI scan) or symptom duration >24 hours. |
| *VHIR-FMT-Barcelona* | Cases were selected through demonstration of acute ischemic stroke in a neuroimaging study during the first 7 days after stroke. |
| *CADISP* | patients with an ischemic stroke without cervical artery dissection (non-CeAD ischemic stroke) were recruited |
| *ARIC* | all local hospitals annually provided lists of stroke discharges (International Classification of Diseases, Ninth Revision, Clinical Modification codes 430 to 438), which were scrutinized for ARIC participant discharges. |
| *JHS* | The definition of stroke was based on the World Health Organization (WHO) criteria for definition of stroke or clinical criteria in which case the WHO criteria might not have been satisfied, but there is clinical evidence sufficient for a diagnosis of stroke to be made. |
| *Helsinki 2000 Ischemic Stroke Genetics Study* | Only patients with positive neuroimaging findings for a newonset brain infarction were recruited following written informed consent. |
| *Hisayama-FSR* | Ischemic stroke was defined as a sudden nonconvulsive, focal neurologic deficit lasting longer than 24 hours due to brain ischemia. The diagnoses of ischemic stroke and its subtypes for all cases were made by stroke neurologists of the hospitals, referring to detailed clinical features and ancillary laboratory examinations. |
| *HVH 1 & 2* | Ischemic stroke cases satisfied one or more of the following criteria: (a) Focal deficit, without evidence of blood on CT or MRI, (b) Focal deficit, with mottled appearance in the appropriate location on CT, or (c) surgery or autopsy evidence of infarction. |
| *INTERSTROKE* | cases were stroke patients with acute first stroke (within 5 days of symptoms onset and 72 hours of hospital admission) in whom neuroimaging (CT or MRI) was performed. Stroke was defined with the WHO clinical criteria for stroke. |
| *MDC* | Criteria for stroke was rapidly developing clinical signs of local or global loss of cerebral function lasting for >24 hours or leading to death before then, with no apparent cause other than cerebral ischemia or hemorrhage. |
| *RACE* | Cases were eligible for inclusion in the study if they: (1) are aged at least 18 years; (2) presented with a sudden onset of neurological deficit affecting a vascular territory with sustained deficit at 24 hours verified by medical attention within 72 hours after onset (onset is defined by when the patient was last seen normal and not when found with deficit); (3) the diagnosis was supported by CT/MRI; and (4) presented with a Modified Rankin Score of < 2 prior to the stroke. |
| *SAHLSIS* | Inclusion criteria was ischemic stroke which was defined as an episode of focal neurological deficits with acute onset and lasting > 24 hours or until death, with no apparent non-vascular cause, and no signs of primary hemorrhage on brain imaging. |
| *SIFAP* | First-ever (80.5%) and recurrent ischemic strokes were included. MRI was a mandatory procedure but, in the case of negative or missing MRI, a qualified stroke neurologist could confirm the clinical diagnosis |
| *SLESS* | One consultant neurologist performed stroke subtyping using data collected on a standard proforma with additional review of all original brain imaging in all patients, as well as review of original notes when necessary. |
| *UK - young lacunar stroke DNA resource* | Lacunar stroke was defined as a clinical lacunar syndrome, with an anatomically compatible lesion on MRI (subcortical infarct ≤15 mm in diameter). |
| *ICH* | Cases were ascertained across participating studies according to predefined standardized criteria. Spontaneous ICH was defined as a new and acute neurological deficit with compatible brain imaging (computed tomography or magnetic resonance imaging) showing the presence of intraparenchymal bleeding. |

**Heart failure**

(Heart Failure Molecular Epidemiology for Therapeutic Targets (HERMES) Consortium, https://www.hermesconsortium.org/, PMID: 29212778)

Cases from following cohorts:

| **Study** | **Definition** |
| --- | --- |
| ARIC | Incident HF: the first HF hospitalization or presence of HF code on death certificate since baseline visit through 2013. ICD-9 code 428.x, and deaths with ICD-9/10 codes of either 428.x or I50.  Prevalent HF: ascertained at first visit. If the participant reported to have taken any medication for heart failure, or qualifies for the Gothenburg Criteria. |
| BIOSTAT-CHF | Based on physician diagnosis, previous documented admission with heart failure requiring diuretic treatment, treatment with furosemide ≥20 mg/day or equivalent. |
| CHS | Identified by self-report or administrative data validated by physician’s review of medical records, as described in previous reports (PMID: 1669507). |
| COGEN | Inclusion criteria: patients ≥18 years, LVEF <40% or symptoms of clinical HF assessed by a physician including NYHA>1 Exclusion criteria: patients with cardiac valvular pathology (e.g. aortic stenosis), HTx, PAH or other structural heart disease |
| deCODE | ICD-10: I50 and subcodes, ICD-9: 428 and subcodes |
| EGCUT | ICD-10: I50 and subcodes. |
| EPHESUS | The presence of pulmonary rales, chest radiography showing pulmonary venous congestion, or the presence of a third heart sound. |
| EPIC-Norfolk | ICD-10: I50 and subcodes |
| FHS | Criteria for defining heart failure in the FHS have been described previously (PMID: 5122894, 16837677). |
| FINRISK | ICD-10: I50, I110, I130 and I132; ICD-9: 4029B, 404, 4148, 428; ICD-7: 42700, 42710, 428 or special drug reimbursement for heart failure medications (requires a medical certificate that meets predefined criteria for heart failure). |
| GoDARTS | Echocardiographic evidence of left ventricular systolic impairment and diuretic prescription or admission to hospital with HF and receipt of a loop diuretic prescription |
| GRADE | Patients who were ≥18 years of age with a diagnosis of at least moderate systolic left ventricular dysfunction (EF ≤30%), and who had an ICD at the University of Pittsburgh Medical Center. |
| LURIC | The combined presence of symptoms of dyspnea on exertion and cardiac disease with impaired left ventricular function. |
| MDCS | Diagnosis codes 427.00, 427.10, and 428.99 for ICD-8, 428 ICD-9, and I50 and I11.0 for the ICD-10 as primary diagnosis, according to a previous validation study (PMID:15916919). |
| PHFS | Diagnosed by a heart failure cardiologist based on clinical evaluation and cardiac imaging |
| PIVUS | ICD codes 427.00, 427.10, 428 (ICD-9), I50 (ICD-10) and hypertensive heart disease with heart failure, I11.0 (ICD-10) PMID:15916919. |
| PREVEND | Heart failure cases were ascertained using criteria in accordance with the Heart Failure Guidelines of the European Society of Cardiology (ESC). |
| PROSPER | Based on a combination of symptoms and signs, including chest radiograph with fluid congestion or echocardiogram with severely diminished LV function. |
| Regeneron/Geisinger | ICD-10: I50 and subcodes. |
| Rotterdam study 1 | Prevalent heart failure at baseline was assessed using a validated score based on the European Society of Cardiology recommendation, identified from hospital discharge diagnoses, and restropective medical records screening. Cases of incident heart failure were obtained by continuously monitoring participants for the occurrence of heart failure during follow-up through general practitioners’ records and hospital discharge diagnoses. |
| SHIP | According to a modified Rotterdam definition (PMID: 10213348). |
| SOLID | Heart failure status at enrolment was identified from medical record with no specific definition. HF during follow up were defined as admission to hospital or attendance at an acute health care facility for administration of intravenous diuretic treatment, escalation of diuretic doses, and/or inotropes. |
| TwinGene | Based on ICD-10: I50; ICD-8 and ICD-9 428 |
| UK Biobank | Individuals with self-reported "HF/pulmonary edema" or "cardiomyopathy"; or ICD-10: I11.0, I13.0, I13.2, I25.5, I42.0, I42.5, I42.8, I42.9, I50.0, I50.1, I50.9; ICD-9: 4254, 4280, 4281, 4289. |
| ULSAM | ICD codes 427.00, 427.10, 428 (ICD-9), I50 (ICD-10) and hypertensive heart disease with heart failure, I11.0 (ICD-10) as possible diagnosis of heart failure. |
| WGHS | Heart failure cases were ascertained by cardiologists from medical records. |
